# The Perfect Storm: A Case of Thyrotoxic Crisis Masking as Altered Mental Status in the South Bronx

**DOI:** 10.7759/cureus.24510

**Published:** 2022-04-26

**Authors:** Samuel Fransen, Tao Do, Mehrdad Alaie

**Affiliations:** 1 Emergency Department, SBH (St. Barnabas Hospital) Health System, New York City, USA

**Keywords:** thyroid storm, thyrotoxicosis, metabolic psychosis, graves´disease, community aquired pneumonia, severe sepsis

## Abstract

Thyrotoxic crisis better known as thyroid storm is a rare life-threatening endocrine emergency that is the result of dysregulated hypermetabolic state caused by excessive production and release of thyroid hormones compromising multiple organ systems. Unfortunately, this condition carries significant mortality depending on whether the thyroid storm was associated with thyrotoxicosis. Early recognition and treatment are paramount to survival. Here we describe a case of thyroid storm complicated by pneumonia and gastrointestinal bleed in which the patient's baseline mental status was restored with prompt identification and treatment and led to a successful recovery.

## Introduction

Thyroid storm is a dangerous and grave complication of thyrotoxicosis. The mortality rates of thyroid storm vary widely and are thought to be between 8-25% despite advancements in its treatment and improvement in supportive treatment [1]. It is a status of dangerously higher levels of metabolic rate in all systems of the body, due to overproduction of the thyroid hormones. Thyroid storm occurs in 1-2% of individuals with hyperthyroidism [2], with Grave’s disease being the most common cause [3]. It can be triggered by a number of other conditions including infections, stress, trauma, congestive heart failure, heart attack, pulmonary embolism, stroke, diabetic ketoacidosis, surgery, intense emotional distress, and of course toxic levels of thyroid hormone taken exogenously. Common symptoms and signs of thyroid storm include the following: a sudden soaring of body temperature, tachycardia, hypertension, vomiting, diarrhea, dehydration, and changes in mental status. These changes in mental state as seen in the patient that is being presented can present as acute agitated delirium or a depressed mental state [4]. 

We present a case of thyroid storm presenting as altered mental status with signs that initially were presenting as sepsis with a source, and during initial resuscitation had little to no history, and only after prompt recognition and treatment for suspected thyrotoxicosis did family arrive with a history consistent with thyroid disease. Prompt recognition of thyroid storm is a clinical diagnosis [5], not a lab result-driven diagnosis, and fast recognition of such led to successful resuscitation of our patient.

## Case presentation

A 60-year-old female presented to the emergency department from home via Emergency Medical Services (EMS) with altered mental status for the past three days. Per EMS patient was accompanied by her daughter and said that the patient had been progressively confused for several days and was increasingly combative. The daughter was unsure of her medical history as a caretaker, who was not present, usually cared for the patient but said the patient saw a hormone doctor who gave her medications. EMS noted that the patient had two small volume episodes of emesis during transport. During the encounter, the patient appeared agitated and responded to all questions by stating “Nothing is wrong; I feel fine.” The remainder of the history was not attainable from the patient. The daughter came to the emergency department with the caretaker several hours after the patient had arrived. Resuscitation and treatment for the patient's suspected thyroid storm had already been in progress by then

**Figure 1:**
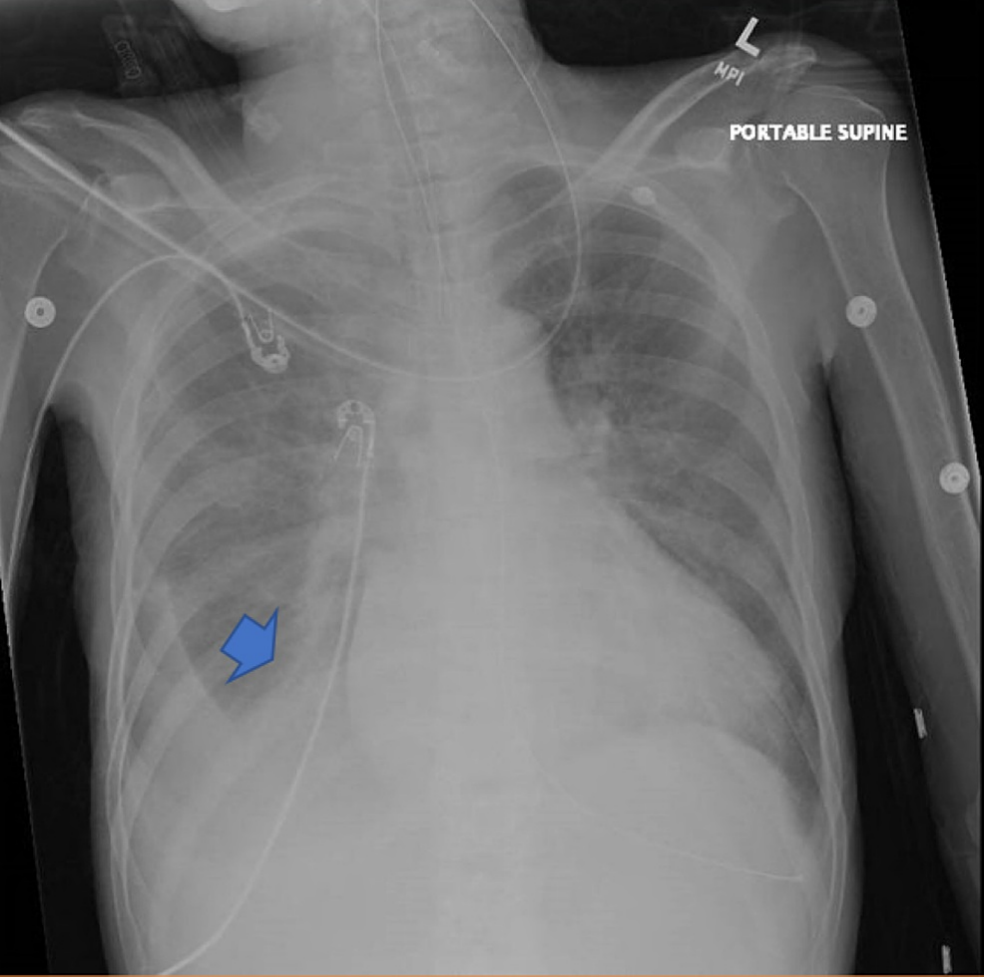
Chest x-ray

Her initial vital signs were concerning for fever (40C), hypotension (94/62), tachycardia (154 beats per minute), and tachypnea (30 breaths per minute) while her O_2_ saturation was 86% on room air. She was thin, disheveled, jaundiced, and toxic-appearing, and was oriented only to name. She had proptotic and icteric eyes. Her cardiopulmonary exam revealed tachycardia with crackles auscultated in mid lung fields with diminished lung sounds at the bases. Her abdomen was mildly tender throughout with hyperactive bowel sounds, but nonrigid or peritonitic. She appeared neuro-vascularly intact, moving all extremities, and had pulses bounding in all four extremities distally. While in the process of finishing the physical exam and getting access to the patient, the patient rapidly became obtunded and did not respond to noxious stimuli. Vital signs were repeated, with worsened hypotension (64/31) and a continued tachycardia (150 bpm). The patient was intubated for airway protection and an orogastric (OG) tube was placed, which produced approximately one liter of coffee ground-appearing content. A chest x-ray showed the endotracheal tube (ETT) at the appropriate depth, OG tube appropriately placed, and bilateral scattered opacities (Figure 1).

EKG, not shown, showed atrial fibrillation. A quick point-of-care ultrasound showed what appeared to be atrial fibrillation with atria contracting too quickly and out of rhythm with the ventricles. No pericardial effusion was appreciated, and no dissection was appreciated. Multiple dynamic air bronchograms seen with scattered B lines were seen. A differential that consisted of rapidly progressing septic shock secondary to pneumonia, upper GI bleed, some type of sympathomimetic toxic ingestion, thyrotoxicosis, ischemic liver disease, and pheochromocytoma. Given the vague history from EMS of the patient seeing a hormone doctor, and the constellation of symptoms and physical exam presentation, thyroid storm was placed higher on the differential. The patient did not respond to one liter of isotonic intravenous fluids, so a second liter was started. Given the point-of-care ultrasound showed no aortic dissection and there was no clear sign of intraabdominal bleeding, surgical emergencies were excluded from the differential. The Burch-Wartofsky criteria [5] is a quantitative diagnostic tool that assigns points for dysfunction of thermoregulatory, GI, cardiovascular, and central nervous systems that can help clinicians stratify the likelihood of thyroid storm in a patient. After calculating a score of >60, which points to thyroid storm being highly likely and the actively decompensating, empiric treatment for thyroid storm began. Per the Burch-Wartofsky, the patient had a score of 60 (delirium=20; nausea, vomiting=10; tachycardia=25; moderate pulmonary edema=10). Treatment for thyroid storm began immediately after intubation and confirmation of the ETT with propranolol 40 mg every eight hours with IV fluids and prompt empiric treatment with broad-spectrum antibiotics covering for the bilateral multi-lobar pneumonia. Then methimazole was given and ordered every eight hours, potassium iodine ordered as well, and high-dose hydrocortisone at 100 mg (IV) every eight hours. ECGs were obtained before and after treatment with propranolol, with a noticeable decrease in heart rate in response to treatment. With the patient still in atrial fibrillation, the ventricular rate decreased to 103. A high-dose proton-pump inhibitor (PPI) infusion was started for GI bleed. Magnesium and potassium were repleted as the patient was found to be hypokalemic. Potassium iodine was given post methimazole.

Significant findings from the evaluation included a hemoglobin of 7.3 g/dL, total bilirubin of 10.7 mg/dL, alkaline phosphatase of 158 U/L, thyroid-stimulating hormone (TSH) 0.01 U/mL, and thyroxine (T4) 29.2 ng/dL (See Table 1). A computerized tomography (CT) scan of the chest and abdomen with contrast demonstrated multi-lobar pneumonia with a right-sided pleural effusion. CT scan of the brain without contrast was unremarkable.

**Table 1:** Initial laboratory results: complete blood count and comprehensive metabolic panel with thyroid-stimulating hormone and thyroxine

Complete Blood Count with differential	Unit	Reference Range
White blood cell count	19.0 × 10^3^/uL	4.0-10.0x 10^3^/uL
Red blood cell count	2.84 × 10^6^/mL	3.93-5.22x 10^3^/uL
Hemoglobin	8.3 gm/dL	11.2-15.7 gm/dL
Hematocrit	24.8%	34.1-44.9%
Platelet count	79 ×10^9^/L	182-369x 10^3^/uL
Comprehensive metabolic panel		
Sodium	148 mEq/L	135-145 mEq/L
Potassium	3.7 mEq/L	3.5-5.3 mEq/L
Chloride	123 mEq/L	96-108 mEq/L
Carbon dioxide	18 mEq/L	23-30 mEq/L
Glucose	130 mg/dL	70-99 mg/L
Blood urea nitrogen	98 mg/dL	8-23 mg/L
Creatinine	3.2 mg/dL	0.6-1.2 mg/dL
Glomerular filtration rate	15 mL/min/1.73m2	60-999 mL/min/1.73m^2^
Calcium	8.9 mg/dL	9.2-11.0 mg/dL
Bilirubin	5.7 mg/dL	0.1-1.2 mg/dL
Aspartate aminotransferase (AST)	26 U/L	4-36 IU/L
Alanine aminotransferase (ALT)	24 U/L	8-33 IU/L
Thyroid-stimulating hormone (TSH)	0.01 U/mL	0.3-5 U/mL
Thyroxine (T4)	29.2 ng/dL	0.8-2.8 ng/dL

It was after this stabilization process that the daughter and caretaker both arrived and provided additional history. The patient had been living with her daughter and the caretaker, who normally takes care of the patient, and her medications had not been present for several days. The patient had a history of hyperthyroidism and had not been taking methimazole. The team had known the patient had a probable endocrinologist she received medications from, and with other obvious endocrinological emergencies ruled out, the patient's presentation was consistent with thyroid storm for which treatment had been started empirically. Endocrinology, Gastroenterology, and Critical Care were consulted for further medical management.

During the hospital course, the patient's pneumonia resolved with appropriate treatment and she was able to undergo endoscopy to evaluate for a GI bleed. Endoscopy showed no active bleeding. After one week after admission, the patient's TSH and T4 began to normalize. Endocrinology resumed her medications and her thyrotoxicosis was resolved. The patient was extubated a week after, resuming oral medications for her hyperthyroidism. The patient ultimately had a complicated stay in the hospital but could be discharged three weeks later with no adverse sequelae.

## Discussion

Thyrotoxicosis is one of the few endocrine emergencies that may present to the emergency department. It has a low incidence rate of 0.57-0.76 cases/100,000 persons in the United States (US) per year, 4.8-5.6/100,000 hospitalized patients per year [6], and an associated high overall mortality rate of 10-30%. Early diagnosis and treatment are vital for this patient population. With the arrival of our patient in a critically ill state with the inability to provide any history, we would like to highlight the importance of relying on multi-system physical exam findings and maintaining a high index of suspicion to promptly achieve the diagnosis.

Four main clinical features that suggest thyroid storm include fever, tachycardia, GI symptoms, and CNS involvement [3]. As stated, our patient was hyperthermic with a temperature of 40^o^C, tachycardic with a heart rate >150, and a recent history of hematemesis. However, despite all the concerning signs and symptoms, the most striking exam finding was the CNS involvement. Upon arrival, the patient was awake, confused, and unable to appropriately engage in conversation. An association of acute psychosis and thyroid storm has been well described in prior literature. In a recent case study by Desai et al. [7], of 28 patients diagnosed with thyroid storm, eight had acute psychosis as a main symptom. As emergency room clinicians, we often see patients presenting in an altered mental status, with differential diagnoses involving substance abuse, acute psychiatric decompensation, infections, metabolic, CNS, etc. However, it is important to note that acute psychosis may be the only overt physical exam finding in a severely ill, thyroid toxicosis patient.

Another physical exam finding worth noting upon arrival was profound scleral icterus, proptosis, and jaundice. Although no medical history was provided on arrival, it was confirmed later, during the hospital stay, that the patient did not have any underlying hepatic-biliary disease and investigatory studies showed total bilirubin at 10.7 (Reference range: 0.1-1.2 mg/dL), Alanine transaminase (ALT) and aspartate aminotransferase (AST) was 14 and 35, respectively (reference range: 4-36 and 8-33 IU/L). Abdominal ultrasound showed gallbladder wall thickening and biliary sludge. It is hypothesized that the cause of cholestasis in thyrotoxicosis includes increased hepatic oxygen consumption without an increase in hepatic blood flow due to a hypermetabolic state, resulting in inefficient bile transport in cholestasis [8]. Despite the unclear etiology, jaundice is an important finding that is often not associated with thyroid disorders and, as such, should be considered as part of the differential diagnosis [8-9]. On chart review, the endoscopy report reported no ulcers or obvious sources of bleeding.

The Burch-Warsofsky point scale quantifies the likelihood of thyroid storm based on different criteria such as thermoregulatory dysfunction, CNS effects, gastrointestinal-hepatic effects, cardiovascular dysfunction, heart failure, and prior history [4]. As always, this scoring system should not be the only tool used to risk-stratify patients. The team had a vague history of the patient following up with an endocrinologist and the exam was consistent with hyperthyroidism; the relevant data was placed on the Burch-Warsofsky point scale and had a total score of 85, which along with clinical suspicion pointed to active thyroid storm. It is critical to recognize and diagnose thyroid storm quickly, as mortality can reach up to 20% in some cases [1], and our case could have been interpreted as severe sepsis due to pneumonia if we had not used the Burch-Wartofsky criteria and had high clinical suspicion.

Thyroid storm should be recognized and treated quickly, with timely empiric treatment. As demonstrated in the current literature, the goals of therapy include not only targeting the synthesis and release of thyroid hormone but also minimizing the effects of circulating hormone and preventing further end-organ damage. Additionally, the order of therapy in treating thyroid storm is important, with thioamide therapy preventing thyroid hormone synthesis prior to iodine therapy to prevent the release of pre-existing hormones [10]. The first step in therapy is preventing impending cardiovascular collapse and the peripheral effect of the thyroid hormone with the use of beta-blockade. In many tissues, hyperthyroidism is associated with an increase in beta-receptor activity [11]. Blockade of the beta-receptors has been one of the cornerstones of therapy for over twenty years [12]. Propranolol is the most commonly used beta-blocker due to its nonselective adrenergic antagonism and ability to decrease the peripheral conversion of T4 to T3 [13]. The second goal of therapy is to inhibit new thyroid hormone synthesis. Utilization of thionamides, such as propylthiouracil (PTU) or methimazole, function by inhibiting thyroid peroxidase, a key enzyme involved in the formation of T3 and T4 from thyroglobulin. The third goal is the inhibition of thyroid hormone release. Iodine administration has been shown to block the release of preformed hormones by inhibiting the proteolytic release of T3 and T4 from thyroglobulin. With all therapies in concert and in this order, it has been demonstrated that serum T4 levels can reach a normal range within four to five days.

Hyperthyroid states that progress to thyrotoxicosis are usually a result of an underlying cause. Thus, a prudent clinician should investigate for reasons as part of the treatment. Precipitating events should be investigated. Previously, thyroid surgery was known to be the common precipitant [10]; however, in the light of preoperative planning and the use of radioactive iodine, it has now become a rare cause. Presently, the most common cause of thyroid storm is infection, as seen in our case. However, there have been many reported precipitating events including surgery, major trauma, iodine exposure from radiocontrast, amiodarone use, and exogenous use of thyroid medications. Searching for precipitating causes with laboratory data and diagnostic imaging is critical once initial treatments are underway. In the case of our patient, she had many potential precipitating causes: lobar pneumonia (as seen on x-ray and CT abdomen), non-adherence with her methimazole, significant upper GI bleed, and anemia. To date, our literature search has shown only three cases of GI bleeding in association with thyrotoxicosis. It may be worth future investigations to find any correlation between the two processes [8].

## Conclusions

Thyroid storm is a difficult, non-specific, condition that emergency department physicians must diagnose in a timely manner. Timely clinical recognition and treatment must be made as mortality increases with delays in treatments. Treatment must be given in concert without waiting for the results of confirmatory tests to decrease mortality. Investigations into precipitating causes of thyroid storm should occur in concert without delay.
